# The Importance of Gestational Sac Size of Ectopic Pregnancy in Response to Single-Dose Methotrexate

**DOI:** 10.1155/2013/269425

**Published:** 2013-05-14

**Authors:** Parichehr Kimiaei, Zahra Khani, Azadeh Marefian, Maryam Gholampour Ghavamabadi, Maryam Salimnejad

**Affiliations:** ^1^Department of Obstetrics and Gynecology, Mahdiyeh Hospital, Shahid Beheshti University of Medical Sciences, No. 16, Fadaieaneslam Street, Shoush Avenue, Tehran 1185817311, Iran; ^2^Department of Obstetrics and Gynecology, Tehran University of Medical Sciences, Tehran 1417614411, Iran

## Abstract

This retrospective cohort study was designed in a selective group of 185 patients diagnosed with and treated for ectopic pregnancy. Intramuscular administration of a single dose of methotrexate (50 mg/m^2^) was performed to measure predictors of failure or resistance to treatment necessitating surgical intervention. During the time of treatment with a single dose of MTX, 20 patients (10.8%) failed to response, in which 6 of 20 (30%) indicated side effects to MTX and rupture of the ectopic pregnancy. Remaining cases (*n* = 14) showed resistance to the drug; the level of **β**-hCG did not fall at least 15% during 7 days after treatment and necessitated laparotomy. In backward-step analysis by multiple logistic regressions of various types of predictor factors, size of gestational sac (coefficient = 1.91, OR = 6.78, 95% confidence interval = 3.18–8.22) and baseline level **β**-hCG (coefficient = 1.60, OR = 5.0, 95% confidence interval = 4.26–6.72) had significant correlation with leading EP patients failing to response to MTX. This study suggests that further investigation for finding relative contraindications of MTX treatment in EP women should be considered on the gestational sac size because other variables are in the causal pathway of this variable.

## 1. Introduction

Two of every 100 pregnancies lead to ectopic pregnancy (EP) because of misplacement of blastocyte outside of the endometrium of the uterus. EP remains as a critical issue in women health during the 21st century [1] especially in the maternal mortality as of first trimester of pregnancy [2, 3].

Medical treatment with local or systemic and multiple or single methotrexate (MTX) is a safe and effective alternative to surgery management of properly selected cases of EP detected by advanced diagnostic tools before rupture [4, 5].

The highest success rate of MTX in the treatment of EP was mentioned in nonlive tubal EP possibly affected by patients' selection criteria and different methods of administration [6–8].

However, previous reports showed that the usage of MTX has different side effects limiting the application of MTX in all patients. 

This study was designed to study the outcome of EP after single intramuscular MTX treatment for finding the relation of clinical and laboratory parameters of cases in the treatment outcome.

## 2. Methods

The study was conducted from 2009 to 2010 after receiving approval from Ethics and Research Committee of Shahid Beheshti University of Medical Sciences and Health Services. Amongst all pregnant women with definitive tubal EP diagnosis (abnormally low *β*-hCG doubling rates less than every 48 hours, together with sonographic identification of a gestational sac outside the uterus), we selected patients who persisted after performing the following exclusion criteria: evidence of immunodeficiency, moderate to severe anemia, leukopenia or thrombocytopenia, sensitivity to MTX, active pulmonary disease, active peptic ulcer disease, clinically important hepatic dysfunction, clinically important renal dysfunction, hemodynamic instability, pelvic tenderness, presence of fetal heart activity on ultrasound or serum progesterone of higher than 45 nmol/L, evidence of tubal rupture of the EP, and gross rectorrhagia.

Informal consent was obtained on the side of all selectees before the study. According to the designed protocol, all the patients received equal doses of MTX as a single 50 mg/m^2^ dose intramuscularly. Demographic data, gestational age, history of previous EP, pelvic inflammatory disease, history of assisted reproductive technology procedures, indicating signs and symptoms, ultrasound results, serum progesterone levels, and the need for hospital admission were documented. The *β*-hCG levels were measured and recorded at baseline and at intervals of 5 to 7 days. Next, serial serum *β*-hCG was obtained biweekly. If *β*-hCG levels failed to decrease at least 15% of baseline during 7 days after the MTX administration, it is assumed as a failure of treatment (resistance to drug). The second MTX injection was not used in this study.

Moreover, all patients were evaluated wistfully for treatment complication and failure by the vital signs, clinical signs, symptoms, and the hematocrit levels in the early period after MTX injection when the evidences of rupture of the EP were presented or surgical interventions were required.

Statistical analysis on collected data was processed by SPSS software (Version 16.0). Chi square analysis was used for binomial parameters and student's *t*-test was used for comparison means parameters between groups. For evaluating independent association of each predictor factors (including size of gestational sac, baseline level *β*-hCG, maternal age, gestational age, and body mass index) with following response of patients to MTX, multiple logistic regressions were used. Differences were considered statistically significant when the *P* value was <0.05. 

## 3. Results

From all registered EP pregnant women (*N* = 259 cases) in our database, 185 cases were included in the study and were valuable for the final analysis according to the exclusion criteria. Patients maternal mean age ±SD was 31.4 ± 8.5 (range: 18–42) years, and the gestational age ±SD was 8.3 ± 2.5 (range: 7–11) weeks at the time of admission. The mean ±SD for body mass index (BMI) of cases was 24.6 ± 3.2 (range: 19–33) kg/cm^2^.

The lowest diagonal length of gestational sac was 13 mm and the highest one was 96 mm (mean ±SD = 37.2 ± 18.6 mm). *β*-hCG initial level was measured in all patients and its mean ±SD was 2550.3 ± 1150.0 (range: 1220–9640) IU/ml.

Follow-up observations of all patients were uneventful, and their vital signs were stable after intramuscular injection. Moreover, the mean ±SD of hematocrit level did not significantly decline from 37.6%  ± 4.1% (before the procedure) to 34.2%  ± 5.0% (3 hours after the procedure) (*P* value = 0.08). During treatment time with MTX single dose, 20 patients (10.8%) failed to response, in which 6 of 20 (30%) had side effects of MTX and rupture of the EP and remaining cases (*N* = 14) had resistance to drug. The *β*-hCG level did not fall at least 15% during 7 days after treatment and necessitated laparotomy.

Based on the findings, patients were divided into two different groups as follows. Group 1: EP patients having well response to MTX treatment and group 2 for EP women who needed surgical interventions after medical treatment fail.

Of these patients, 42 cases (35.5%) became pregnant after the use of assisted reproductive technology and from these patients 9 cases (21.4%) had failed to MTX treatment. No patients had experienced EP previously, and 5 cases had a known history of pelvic inflammatory disease (PID). The rate of failing to treatment did not rise in patients who had previous PID history.

Comparing highest diameter mean ±SD for EP mass between two groups was meaningfully different (group 1 = 36.3 ± 14.6 versus group 2 = 44.8 ± 19.1; *P* value = 0.036).

The mean ±SD baseline level of *β*-hCG was 2335.8 ± 850.2 in group 1 which was significantly lower than group 2 (3925.1 ± 1810.0; *P* value = 0.002).

Differences in maternal and gestational age as well as BMI and number of gravid between two groups were not statistically significant (Table 1). Moreover, in backward-step analysis by multiple logistic regressions of various types of predictor factors, size of gestational sac (coefficient = 1.91, OR = 6.78, 95% confidence interval = 3.18–8.22) and baseline level *β*-hCG (coefficient = 1.60 OR = 5.0, 95% confidence interval = 4.26–6.72) had significant correlation with leading EP patients failing to response to MTX.

## 4. Discussion

Trophoblast is a rapid proliferative tissue of ectopic embryo which is very sensitive to inhibitory methotrexate (MTX) effect or other folic antagonists on synthesis of thymidylate, serine, methionine, and consequently synthesis, repair, and cellular replication of DNA [9]. 

Therefore, MTX with minimal maternal dose above 10 mg. per week or a single dose of 50 mg/m^2^ and a half-life of 8 to 15 hours is teratogenic or lethal to all human body embryos between 6 and 8 weeks after conception [10, 11]. 

The time in which EPs were completely resolved ranged from 7–43 days after MTX treatment [12, 13]. 

According to the mentioned mechanism, MTX is usually used to obstetric or gynecologic conditions including first-trimester terminations, gestational trophoblastic disease, and EP [14].

The use of a single-dose MTX as a treatment option for EP was introduced 20 years ago by Stovall et al. [15] and reviewed by Feldkamp and Carey [10]. 

During administration, different rates of success were achieved in different studies including 73% reported by Tawfiq et al. [12], 75% reported by Corsan et al. [16], 78% reported by Stika et al. [8] 85% reported by Potter et al. [17], 85.7% reported by Glock et al. [18], 90% reported by Lipscomb et al. [19], and 94.2% reported by Stovall and Ling [20]. According to slick limiting criteria for including patients in the current study, we reported the success rate of 89.2% which was in the range of previous reports in our region [21]. 

Despite this discrepancy, today prognostic factors for successful response to MTX treatment in EP cases had limited reports. This study indicates that the size of gestational sac from imaging findings and the baseline of *β*-hCG level from laboratory finding could predict the future consequences of treatment. These findings are in consistency with previous studies which reported the positive predictive value of factors including *β*-hCG level > 4000 IU/L (65% failure), presence of pelvic pain (56% failure) or vaginal bleeding (53% failure), fetal heart motion on ultrasound, and the size of the EP > 3.5 cm. All predictive factors which were mentioned in this study and similar investigations could be summarized in one cause and effect strings. By this theory, the size of gestational sac had the first part of this string consequently related with the level of *β*-hCG production and falling progesterone levels as a result of vaginal bleeding. Moreover, the size of sac might be predicted by the level of invasion to the serosa and the experience of abdominal pain in cases. However, the cut of point selection for the size of gestational sac used in MTX treatment had different results from various studies. 

This study suggests that further investigation to find relative contraindications should be considered on the mass size because other variables are in the causal pathway.

Surprisingly, no major (neutropenia, oral pain, stomatitis, pneumonia, and cystitis) and minor (nausea and vomiting) side effects of MTX treatment were recorded in our study. 

## 5. Conclusion

It can be concluded that a single-dose MTX is an important option of treatment in EP cases without noticeable side effects like multiple dose injection; however, it should be considered in more studies about the topic with appropriate sample size. 

## Figures and Tables

**Table 1:** Comparison of predictor factors of response to a single dose of methotrexate in 185 ectopic pregnant women.

Parameters	Group 1 (response to treatment)	Group 2 (failure to treatment)	*P* value
Age ± SD (years)	31.1 ± 9.2	33.9 ± 8.3	0.65
BMI ± SD (kg/m^2^)	24.3 ± 2.9	26.7 ± 3.4	0.57
Gestational age ± SD (weeks)	6.9 ± 1.5	8.0 ± 1.7	0.38
Mass size ± SD (mm)	36.3 ± 14.6	44.8 ± 19.1	0.036*
Number of gravid ± SD	2.1 ± 1.5	2.7 ± 1.2	0.45
*Β*-hCG ± SD (IU)	2335.8 ± 850.2	3925.1 ± 1810.0	0.002*

*Significant difference between two groups.
